# Polysomnography validation of SANSA to detect obstructive sleep apnea

**DOI:** 10.3389/fneur.2025.1592690

**Published:** 2025-06-16

**Authors:** Cathy Goldstein, Hamid Ghanbari, Surina Sharma, Nancy Collop, Andrew Namen, Douglas B. Kirsch, Michael Drucker, Rami Khayat, Mark Pollock, Brennan Torstrick, Colleen Walsh, Emily Herreshoff, David S. Frankel, Ilene M. Rosen

**Affiliations:** ^1^Department of Neurology, University of Michigan, Ann Arbor, MI, United States; ^2^Division of Cardiovascular Medicine, University of Michigan, Ann Arbor, MI, United States; ^3^Emory Sleep Center, Emory University, Atlanta, GA, United States; ^4^Atrium Wake Forest Baptist, Winston-Salem, NC, United States; ^5^Atrium Health Sleep Medicine, Charlotte, NC, United States; ^6^Novant Health Cardiology, Winston-Salem, NC, United States; ^7^Division of Pulmonary, Allergy, and Critical Care, Penn State Health and the Sleep Research and Treatment Center, Pennsylvania State University, Hershey, PA, United States; ^8^Pulmonary & Sleep Specialists, Atlanta, GA, United States; ^9^Huxley Medical, Inc., Atlanta, GA, United States; ^10^Division of Sleep Medicine, Perelman School of Medicine, University of Pennsylvania, Philadelphia, PA, United States; ^11^Division of Cardiovascular Medicine, Perelman School of Medicine, University of Pennsylvania, Philadelphia, PA, United States

**Keywords:** sleep disordered breathing, sleep apnea, cardiac arrhythmia, atrial fibrillation, home sleep apnea testing, wearable diagnostic devices

## Abstract

**Study objectives:**

Evaluate the performance of a novel home sleep apnea test with embedded ECG (SANSA, Huxley Medical, Inc.) in the diagnosis of obstructive sleep apnea (OSA).

**Methods:**

This prospective multicenter validation study included 340 participants who underwent simultaneous polysomnography (PSG) and SANSA recordings across 7 clinical sites. Participants were diverse across age, sex, race, skin tone, and body mass index. Diagnostic performance was assessed with the apnea-hypopnea index (AHI) using both Rule 1A and Rule 1B across standard cutoffs for mild, moderate, or severe (≥5 events/h), moderate-to-severe (≥15 events/h), and severe (≥30 events/h) OSA. The agreement for AHI and total sleep time (TST) between SANSA and consensus PSG scores from three independent scorers was evaluated using Pearson’s correlation and Bland–Altman analysis. Sensitivity and specificity were calculated at each OSA severity level. Performance of participating site PSG scores were also evaluated against consensus PSG scores for comparison.

**Results:**

SANSA demonstrated excellent agreement with PSG for most performance parameters. AHI correlation was 0.91 (95% CI: 0.89, 0.93) using Rule 1B and 0.90 (95% CI: 0.87, 0.92) using Rule 1A. Compared to consensus scored PSG, the device detected moderate-to-severe OSA using Rule 1B (the primary endpoint) with a sensitivity of 88% (95% CI: 81, 93%) and specificity of 87% (95% CI: 82, 91%), while site PSG sensitivity was 89% (95% CI: 82, 94%) and specificity was 93% (95% CI: 88, 96%). SANSA TST highly correlated with PSG TST (*R* = 0.82, 95% CI: 0.78, 0.85) and classified sleep epochs with an accuracy of 87.2% (95% CI: 87.0, 87.5%).

**Conclusion:**

The SANSA home sleep apnea test demonstrated robust diagnostic performance for OSA detection including measurement of sleep compared to PSG. Its patch morphology and embedded ECG confer ease of use and multi-diagnostic potential in sleep medicine and cardiology for the detection of OSA and cardiac arrhythmias across diverse clinical populations.

**Clinical trial registration:**

[https://www.clinicaltrials.gov/study/NCT06070389], identifier [NCT06070389]

## 1 Introduction

Obstructive sleep apnea (OSA) is characterized by repeated upper airway obstruction that leads to intermittent hypoxia, intrathoracic pressure swings, sympathetic activation, and arousal from sleep. Patients most commonly seek care for excessive daytime sleepiness, but OSA is also associated with excess mortality and increased risk of cardiovascular disease. The compounded effects of OSA are particularly implicated in atrial fibrillation and heart failure, where disruption of the atrial substrate and adverse hemodynamics contribute to disease progression and recurrence (1–10). Despite its high prevalence and comorbidity, most of the nearly 1 billion people with OSA worldwide remain undiagnosed and less than one in 50 undergo testing, highlighting the need for more efficient and versatile diagnostic tools (11–13).

Polysomnography (PSG) is the gold standard for OSA diagnosis, but it is high cost and requirement of a sleep laboratory staffed by polysomnographic technologists limit accessibility; additionally, insurers are increasingly denying coverage for in lab PSG. Currently available home sleep apnea tests (HSATs) are more readily available (14) but prone to user error and discomfort, which can affect data quality and completeness, resulting in repeat testing and diagnostic delays (15, 16). Moreover, most HSATs only measure cardiac parameters in the periphery with photoplethysmography (PPG) and lack gold standard electrocardiogram (ECG); therefore, HSATs fail to provide actionable insights into cardiovascular conditions comorbid with OSA, such as arrhythmia assessment or hemodynamic parameters to aid in heart failure management. To address these limitations, the SANSA device (Huxley Medical, Inc., Atlanta, GA) was developed as a simple, single-point-of-contact chest patch with multi-diagnostic capabilities (17). The device records 10 physiological channels to diagnose and monitor OSA—including ECG for atrial fibrillation and arrhythmia detection—and can also measure hemodynamic parameters associated with heart failure progression (18). This comprehensive, multi-diagnostic approach could enable simultaneous detection of OSA and cardiovascular comorbidities.

Previous work has reported the design and development of SANSA and its automated algorithm to detect OSA (17). The goal of the current study is to compare SANSA’s diagnostic performance to gold-standard in laboratory PSG in a large, prospective, multicenter cohort.

## 2 Methods

### 2.1 Study design and participants

Across seven American Academy of Sleep Medicine (AASM) accredited clinical sites, individuals undergoing PSG for suspected OSA were also monitored simultaneously with the SANSA device during a single night of recording. This study was approved by a central institutional review board, conformed to the Declaration of Helsinki, and was registered on ClinicalTrials.gov (NCT06070389).

### 2.2 Inclusion and exclusion criteria

Enrolled patients were known or suspected to have OSA based on an assessment by, or under the direction of, a board-certified sleep specialist. Typical symptoms included excessive daytime sleepiness, habitual loud snoring, witnessed apneas, or gasping during sleep. Participants were eligible if they were at least 18 years of age, able to provide informed consent, and willing to undergo simultaneous data collection with the SANSA device and PSG. Consistent with diagnostic clinical practice guidelines for HSAT, exclusion criteria included diagnosed hypoventilation, severe chronic obstructive pulmonary disease (COPD), significant non-respiratory sleep disorders (narcolepsy or parasomnias with sleep behavior that was injurious, erratic, or could lead the subject to leave the bed), neuromuscular disorders associated with respiratory muscle weakness, chronic supplemental oxygen use, pregnancy, and the presence of cardiac implantable electronic devices or severe congestive heart failure (less than 45% ejection fraction) (19). Participants were also excluded for the presence of chest deformities interfering with sensor placement, skin injuries, and adverse reactions to medical-grade adhesives.

### 2.3 Clinical procedures

Participants were screened for eligibility by physician investigators during routine clinic visits or from the PSG schedule on enrollment days based on coordinator and device availability. Prior to overnight recording in the sleep laboratory, written informed consent was obtained, vital signs were recorded, and questionnaires assessing sleep-related symptoms and risk factors, including the STOP-BANG, DOISNORE50, and Epworth Sleepiness Scale were obtained (20–22). PSG and SANSA recordings were collected simultaneously during a single overnight recording in sleep laboratories accredited by AASM. PSG signals were acquired in accordance with the technical specifications described in the AASM Manual for the Scoring of Sleep and Associated Events (Version 2.6) (23). PSG channels included oximetry, pulse rate, airflow via thermistor and nasal pressure transducer sensors, thoracic and abdominal respiratory inductance plethysmography (RIP THO and RIP ABD), electroencephalography (EEG), electromyography (EMG), electrocardiography (ECG), and electrooculography (EOG). Data collection utilized either the Alice (Philips-Respironics, Pittsburgh, PA) or Grael (Compumedics, Charlotte, NC) PSG systems.

Each PSG study was manually scored by three independent Registered Polysomnographic Technologists (RPSGTs) from a core scoring laboratory to ensure a robust ground truth PSG score with known interscorer reliability. Each PSG study was also scored by a scorer at the local participating site. All scoring followed the AASM Manual for Scoring of Sleep and Associated Events (Version 2.6), employing Rule 1A (3% desaturation or arousal) and 1B (4% desaturation) criteria to score hypopneas. SANSA raw data was processed using only the automated scoring algorithm without review by a sleep technologist or physician. The severity of sleep apnea was categorized using standard clinical cutoff thresholds: AHI ≥ 5 for mild, moderate, or severe OSA; AHI ≥ 15 for moderate-to-severe OSA; and AHI ≥ 30 for severe OSA. Only the diagnostic portions of split-night polysomnograms were included in analysis. The three scores from the core laboratory RPSGTs were used to establish a consensus PSG score that was used as the ground truth for all analyses. For continuous measures of AHI and total sleep time (TST), the consensus PSG score was defined as the mean of the three core RPSGT scores. For categorical measures of diagnostic classifications, the consensus PSG score was defined as agreement between 2 out of 3 individual scorers on a classification (e.g., if 2 of 3 scorers classified a participant as positive for a particular severity condition then the participant was positive for that condition). Consensus scoring for epoch-level sleep/wake classification was determined using a similar approach.

### 2.4 Statistical analysis

SANSA and participating site PSG results were both compared to consensus PSG scores as the common reference. Agreement for the continuous parameters AHI and TST was evaluated using Pearson’s correlation coefficient and Bland–Altman limits of agreement (24). The diagnostic performance of SANSA and participating site PSG scores was assessed by calculating sensitivity, specificity, positive predictive value (PPV), negative predictive value (NPV), and overall accuracy at AHI cutoff thresholds of 5, 15, and 30 events per hour using both Rule 1A and 1B. Epoch-level agreement for sleep/wake classification was evaluated using sensitivity, specificity, and accuracy to detect sleep epochs. Interscorer reliability between the three core PSG scores was assessed using the intraclass correlation coefficient, ICC (A,1) (25). The study sample size was selected to test if SANSA’s sensitivity and specificity to detect moderate-to-severe OSA (Rule 1B) were both at least 80%. Sample size was determined using the exact binomial test with one-sided alpha level of 0.025 and overall power of 0.85 to evaluate both endpoints. The resulting analysis required enrollment of at least 310 evaluable datasets. Consistent with PSG validation studies of other HSAT devices, records were pre-specified for exclusion from analysis if SANSA or PSG data were non-interpretable or constituted technical failure; which included equipment detachment or interference, less than 2 h of PSG diagnostic recording time, less than 90 min of PSG TST or 60 min of SANSA TST, and insufficient signal overlap between PSG and SANSA when excluded periods accounted for 30 % or more of total recording time (25–27). Records were also excluded if the participant withdrew from the study, administrative errors precluded analysis, or the device manufacturer’s instructions were not followed. Differences in correlation coefficients were evaluated using a z-test on Fisher z-transformed coefficients (28). Fisher’s exact tests were used to evaluated differences in proportions (29). All statistical analyses were performed in Python (3.11.6).

## 3 Results

### 3.1 Study population

**Figure 1:**
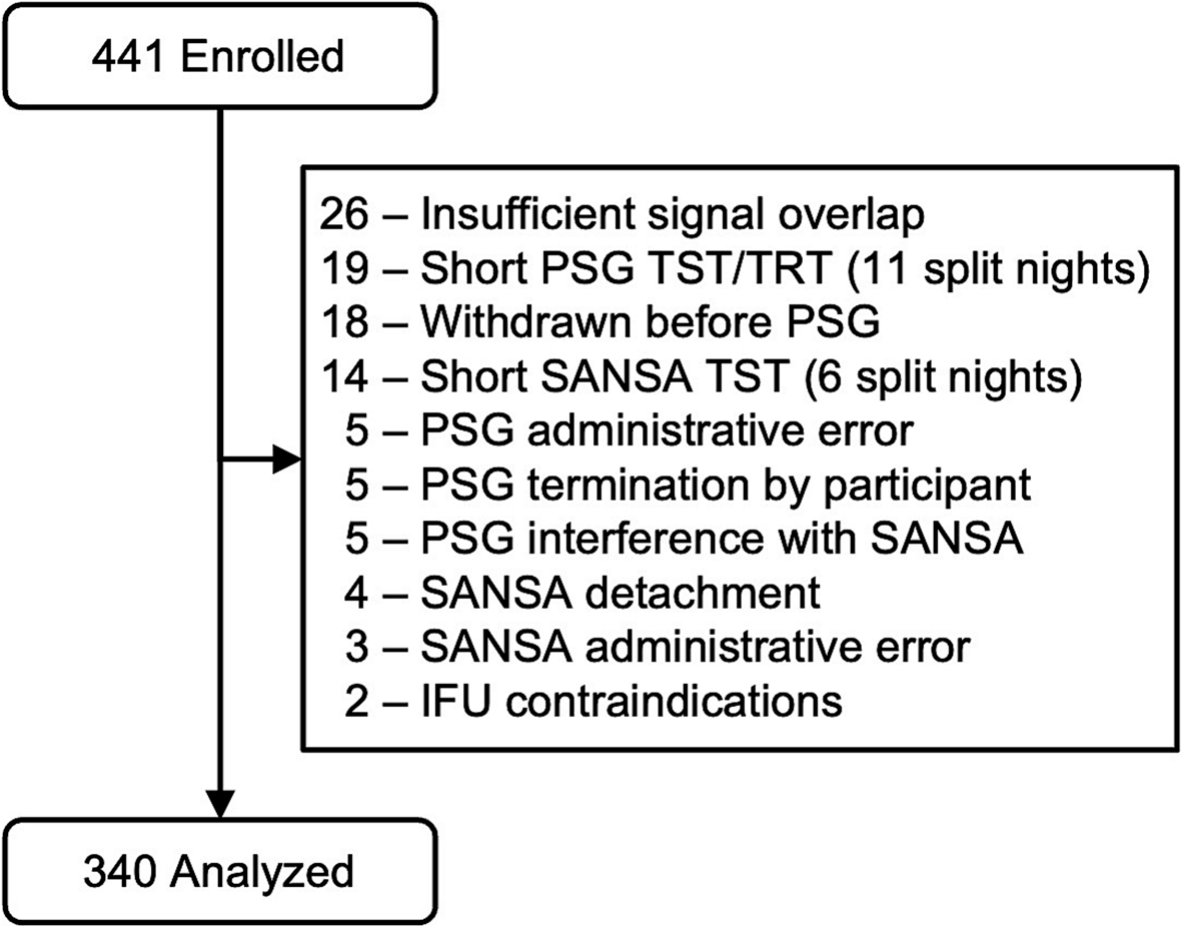
Consort diagram.

A total of 441 participants were enrolled following the inclusion and exclusion criteria, of which 340 were included in the final analysis group. Figure 1 reports record exclusion for pre-specified reasons after enrollment, as described in the methods above. Of the 340 records in the final analysis, participating site PSG scores were unavailable for 160 using Rule 1A and 24 using Rule 1B because the collecting site only scored using one criterion due to local payer and clinic policy. These datasets were excluded from comparisons involving PSG site scores.

Participants were predominantly middle aged (mean 55.4, SD 15.3) and overweight (mean BMI 33.5, SD 8.7); both sexes were well represented (46.8% male, 53.2% female) (Table 1). Participant race was predominantly white (67.9%) and black (27.4%). All categories of the Fitzpatrick skin tone scale were included with lighter skin tones (1–3) representing 74% of participants and darker skin tones (4–6) representing 26% (30). STOP-BANG (mean 4.3, SD 1.7), DOISNORE50 (mean 5.5, SD 1.6), and Epworth Sleepiness Scale scores (mean 9.0, SD 5.1) were consistent with OSA. Medical histories were representative of OSA populations, with a higher prevalence of hypertension (34.1%), asthma (17.4%), diabetes (15.9%), insomnia (8.8%), and arrhythmias (8.6%). A total of 16 recordings were excluded for insufficient sleep time that were not split-night studies (eight due to short sleep time on PSG and eight due to short sleep time on SANSA). These participants were similar in their medical histories compared to the analysis population and a typical sleep disordered breathing clinical population. Two adverse events were reported for skin reaction to the medical adhesive without sequelae.

**Table 1:** Patient demographics.

Variable	
Age (Mean, SD)—yr	(55.4, 15.3)
Male (N, %)	(159, 46.8)
Race (N, %)	
American Indian/Alaska Native	(1, 0.3)
Asian	(5, 1.5)
Black	(93, 27.4)
Native Hawaiian/Other Pacific Islander	(0, 0)
White	(231, 67.9)
Other	(10, 2.9)
Missing	(0, 0)
Race (N, %)	
Hispanic	(14, 4.1)
Non-Hispanic	(326, 95.9)
Weight (Mean, SD)—kg	(97.2, 26.7)
BMI (Mean, SD)—kg/m^2^	(33.5, 8.7)
Fitzpatrick Scale (Mean, SD)	(2.4, 1.4)
STOP-BANG Score (Mean, SD)	(4.3, 1.7)
DOISNORE50 (Mean, SD)	(5.5, 1.6)
Epworth Sleepiness Scale (Mean, SD)	(9.0, 5.1)

### 3.2 AHI

**Figure 2:**
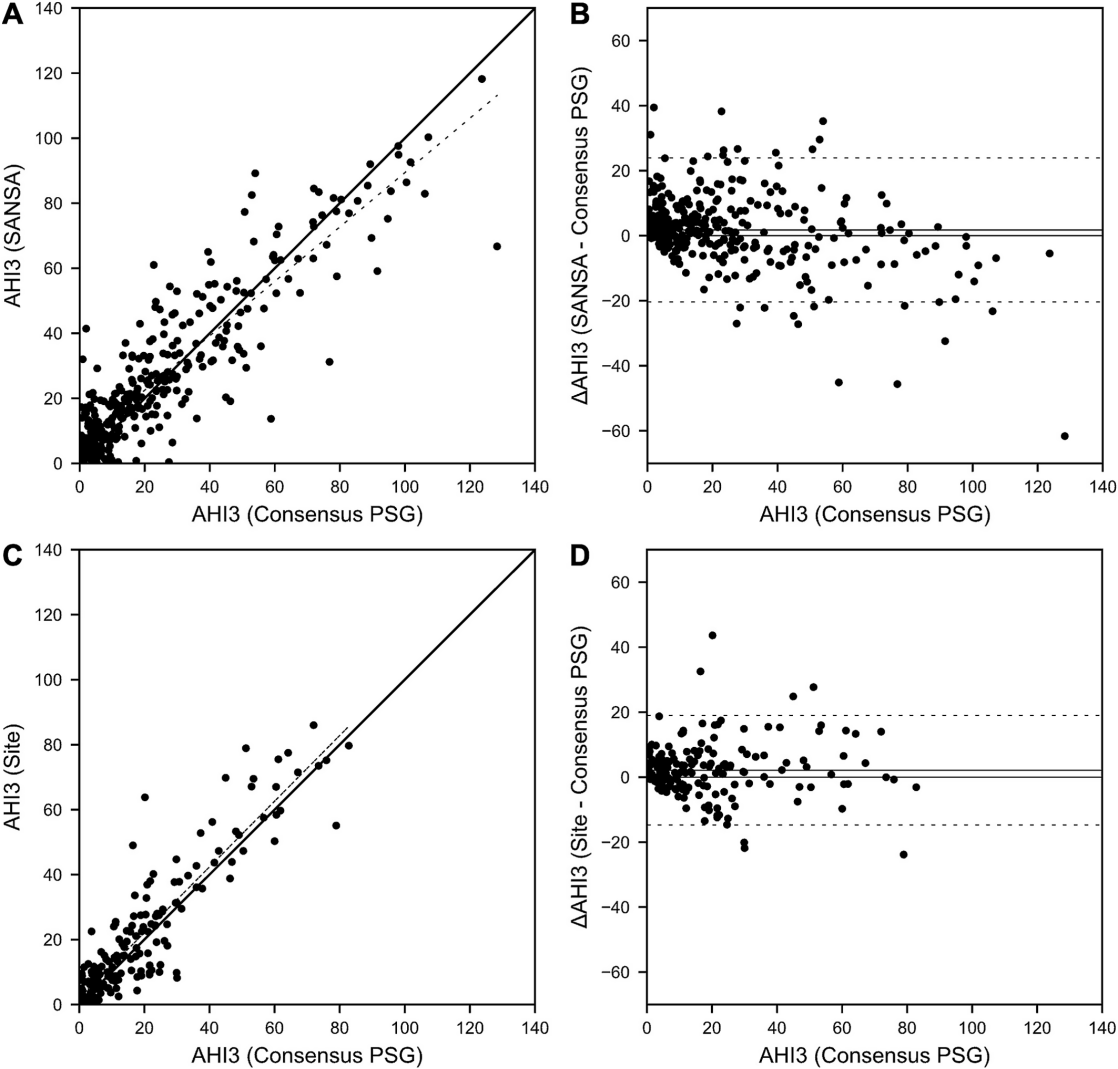
SANSA AHI-3% Correlation and Bland–Altman comparison against PSG. AHI correlation **(A)** and Bland–Altman **(B)** plots of the SANSA multi-diagnostic device compared to the PSG consensus score. Corresponding plots are also shown for PSG site scores compared to PSG consensus score **(C,D)**.

**Figure 3:**
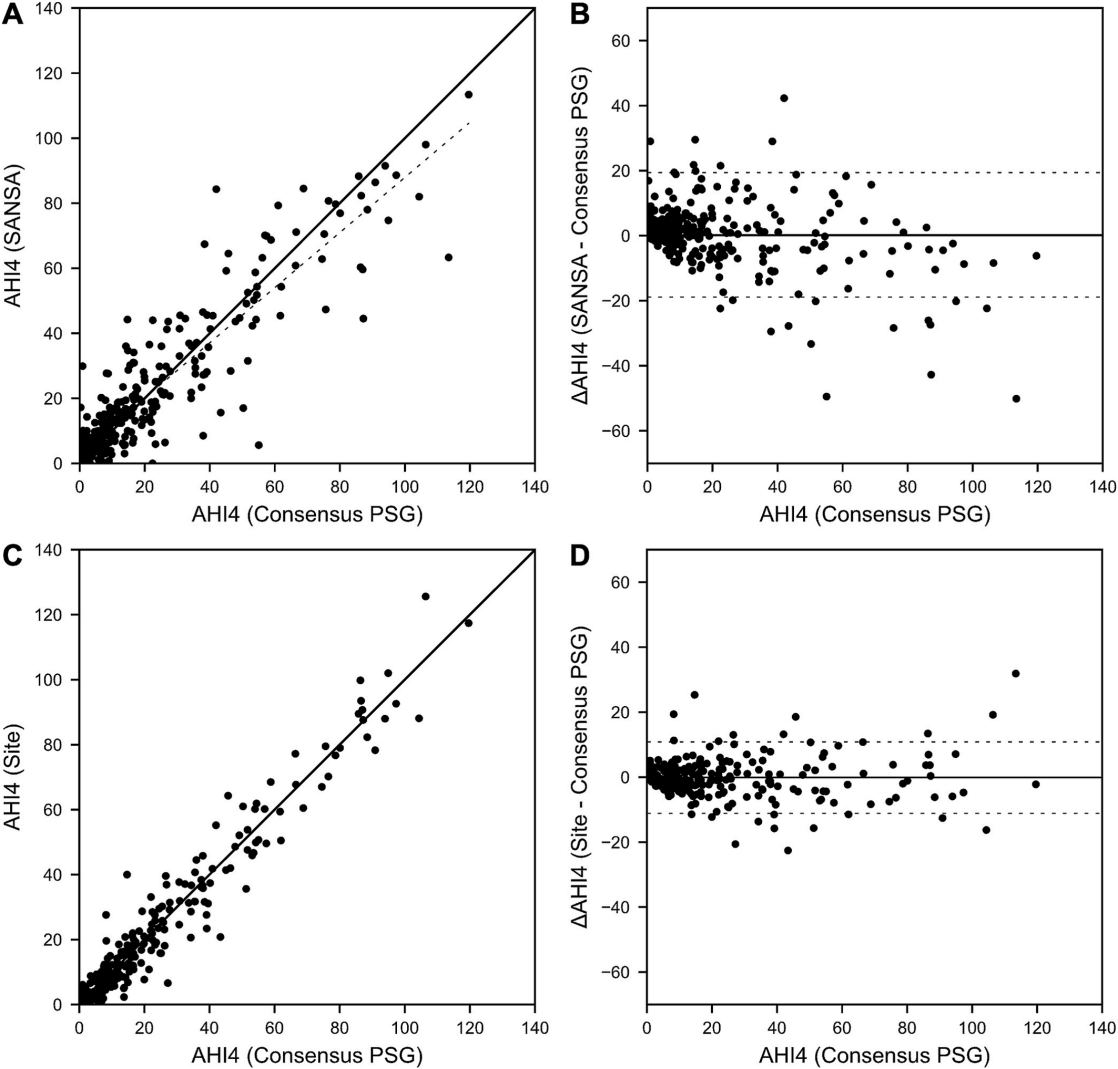
SANSA AHI-4% Correlation and Bland–Altman comparison against PSG. AHI correlation **(A)** and Bland–Altman **(B)** plots of the SANSA multi-diagnostic device compared to the PSG consensus score. Corresponding plots are also shown for PSG site scores compared to PSG consensus score **(C,D)**.

**Figure 4:**
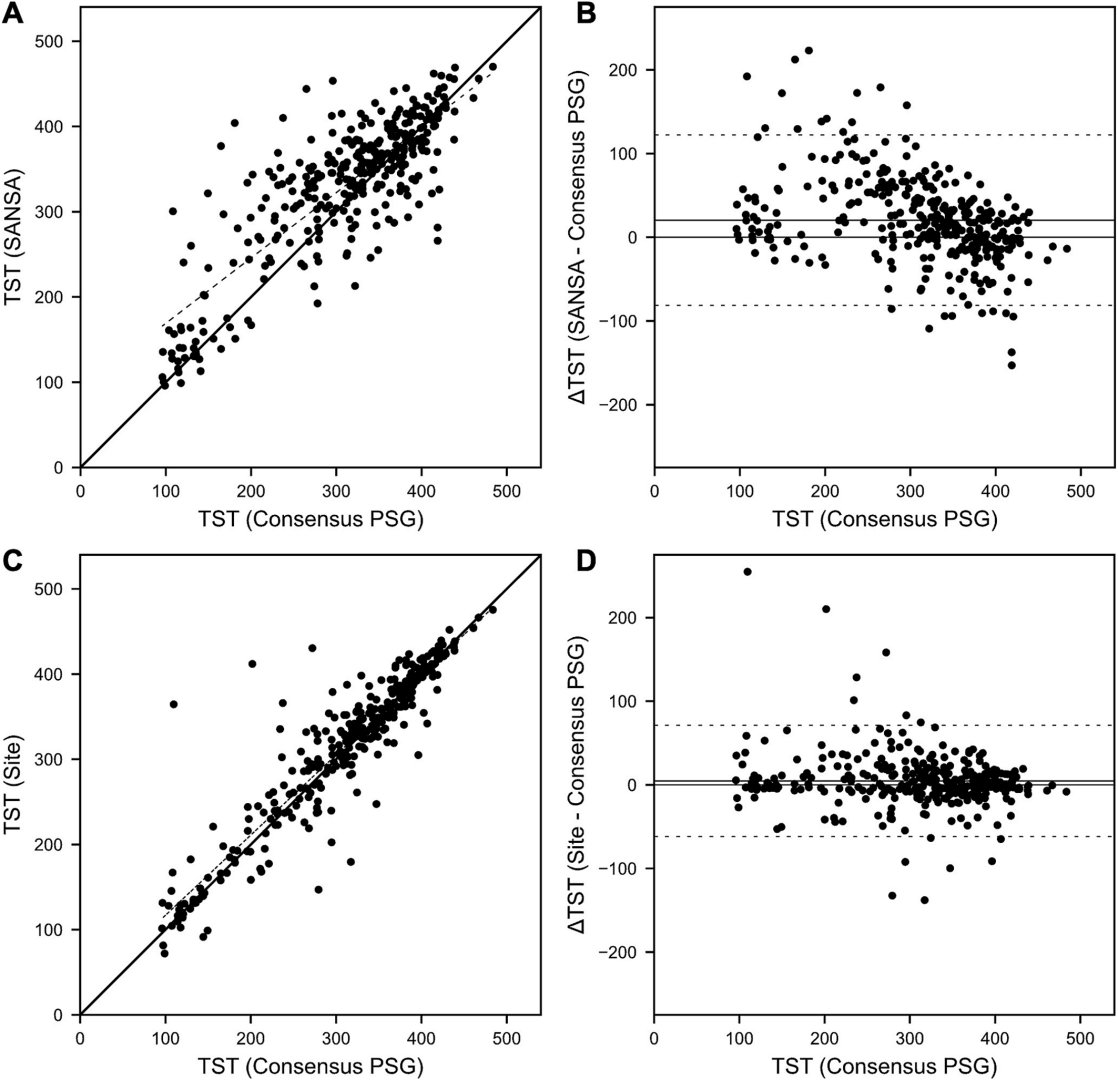
SANSA TST Correlation and Bland–Altman comparison against PSG. TST correlation **(A)** and Bland–Altman **(B)** plots of the SANSA multi-diagnostic device compared to the PSG consensus score. Corresponding plots are also shown for PSG site scores compared to PSG consensus score **(C,D)**.

Results for AHI agreement and diagnostic performance are detailed in Table 2 and Figures 2–4. AHI correlation with consensus PSG was strong for the SANSA device with the use of Rule 1A (*R* = 0.90) and Rule 1B (*R* = 0.91), which was consistent with that of participating site PSG scores. AHI bias compared to consensus PSG was also consistent between SANSA and site PSG using both criteria, yet the limits of agreement were narrower for site PSG.

**Table 2:** AHI endpoints.

		Rule 1A		Rule 1B	
		SANSA	Site PSG	SANSA	Site PSG
Correlation, R		0.90 (0.87, 0.92)	0.88 (0.84, 0.91)	0.91 (0.89, 0.93)	0.96 (0.94, 0.96)^*^
Bias		1.8 (0.6, 3.0)	1.6 (0.1, 3.1)	0.3 (−0.8, 1.3)	−0.4 (−1.2, 0.4)
Lower LoA		−20.4 (−22.5, −18.3)	−18.0 (−20.5, −15.4)	−18.9 (−20.7, −17.1)	−14.6 (−16.0, −13.2)
Upper LoA		23.9 (21.8, 26.0)	21.2 (18.7, 23.7)	19.4 (17.6, 21.2)	13.8 (12.4, 15.2)
Cutoff AHI 5	Se	0.96 (0.93, 0.98)	0.95 (0.89, 0.98)	0.94 (0.90, 0.96)	0.94 (0.90, 0.97)
	Sp	0.55 (0.44, 0.65)	0.53 (0.38, 0.67)	0.71 (0.62, 0.79)	0.88 (0.81, 0.94)^*^
	PPV	0.85 (0.81, 0.89)	0.84 (0.77, 0.89)	0.86 (0.81, 0.90)	0.94 (0.89, 0.97)^*^
	NPV	0.85 (0.73, 0.93)	0.79 (0.62, 0.91)	0.86 (0.77, 0.92)	0.89 (0.82, 0.94)
	Acc	0.85 (0.81, 0.89)	0.83 (0.76, 0.88)	0.86 (0.82, 0.89)	0.92 (0.89, 0.95)^*^
Cutoff AHI 15	Se	0.93 (0.88, 0.96)^*^	0.79 (0.69, 0.87)	0.88 (0.81, 0.93)	0.89 (0.82, 0.94)
	Sp	0.74 (0.67, 0.81)	0.85 (0.77, 0.92)^*^	0.87 (0.82, 0.91)	0.93 (0.88, 0.96)
	PPV	0.80 (0.73, 0.85)	0.83 (0.73, 0.90)	0.81 (0.73, 0.87)	0.88 (0.81, 0.94)
	NPV	0.90 (0.84, 0.95)	0.82 (0.73, 0.89)	0.93 (0.88, 0.96)	0.93 (0.89, 0.96)
	Acc	0.84 (0.79, 0.88)	0.82 (0.76, 0.88)	0.88 (0.84, 0.91)	0.91 (0.88, 0.94)
Cutoff AHI 30	Se	0.87 (0.78, 0.93)	0.97 (0.85, 1.00)	0.82 (0.71, 0.90)	0.91 (0.81, 0.97)
	Sp	0.89 (0.84, 0.93)	0.95 (0.90, 0.98)^*^	0.95 (0.92, 0.97)	0.98 (0.95, 0.99)
	PPV	0.76 (0.67, 0.83)	0.83 (0.69, 0.93)	0.82 (0.71, 0.90)	0.91 (0.81, 0.97)
	NPV	0.94 (0.90, 0.97)	0.99 (0.96, 1.00)^*^	0.95 (0.92, 0.97)	0.98 (0.95, 0.99)
	Acc	0.88 (0.84, 0.91)	0.96 (0.91, 0.98)^*^	0.92 (0.89, 0.95)	0.96 (0.93, 0.98)^*^

This table displays AHI-related endpoints and their 95% confidence intervals (Clopper-Pearson) for SANSA and site PSG using Rule 1A and Rule 1B. An asterisk indicates significantly greater performance for the same scoring rule (*z*-test on Fisher z-transformed correlation coefficients and Fisher’s exact test for proportions). Acc, accuracy; AHI, apnea–hypopnea index; NPV, negative predictive value; PPV, positive predictive value; PSG, polysomnography; Se, sensitivity; Sp, specificity.

Overall, across AHI cutoff thresholds for Rule 1A and 1B, both SANSA and participating site PSG maintained high sensitivity to detect OSA, with slightly lower specificity at lower AHI cutoffs. Using Rule 1B, SANSA detected moderate-to-severe OSA with 88% (95% CI: 81, 93%) sensitivity and 87% (95% CI: 82, 91%) specificity, which was consistent with site PSG performance (versus consensus PSG) of 89% (95% CI: 82, 94%; *p* = 0.843) sensitivity and 93% (95% CI: 88, 96%; *p* = 0.071) specificity. For mild, moderate, or severe OSA, SANSA demonstrated 94% (95% CI: 90, 96%) sensitivity and 71% (95% CI: 62, 79%) specificity, which was consistent with site PSG sensitivity of 94% (95% CI: 89, 97%; *p* = 1.000) but lower than site PSG specificity of 88% (95% CI: 81, 94%; *p* = 0.002).

Using Rule 1A, SANSA detected moderate-to-severe OSA with 93% (95% CI: 88, 96%) sensitivity and 74% (95% CI: 67, 81%) specificity, which was greater than site PSG sensitivity of 79% (95% CI: 69, 87%; p = 0.002) but lower than site PSG specificity of 85% (95% CI: 77, 92%; *p* = 0.043). For mild, moderate, or severe OSA, SANSA demonstrated 96% (95% CI: 93, 98%) sensitivity and 55% (95% CI: 44, 65%) specificity, which was consistent with site PSG performance of 95% (95% CI: 89, 98%; *p* = 0.427) sensitivity and 53% (95% CI: 38, 67%; *p* = 0.862) specificity.

Inter-scorer variability of AHI between the three individual core consensus scorers represented by the intraclass correlation coefficient was 0.92 for Rule 1A and 0.95 for Rule 1B.

To assess potential bias from exclusions, results were also reanalyzed with 35 of the 101 pre-specified exclusions reincluded—representing all excluded records for which both SANSA and PSG scores were available for comparison. These 35 studies were originally excluded for insufficient signal overlap (n = 26), short PSG TST or TRT (*n* = 2), PSG interference with SANSA (*n* = 5), and IFU contraindications (*n* = 2). Re-inclusion of these records did not significantly impact performance for both Rule 1A and Rule 1B compared to the dataset containing all pre-specified exclusions (Supplementary Table S1).

### 3.3 TST

**Figure 5:**
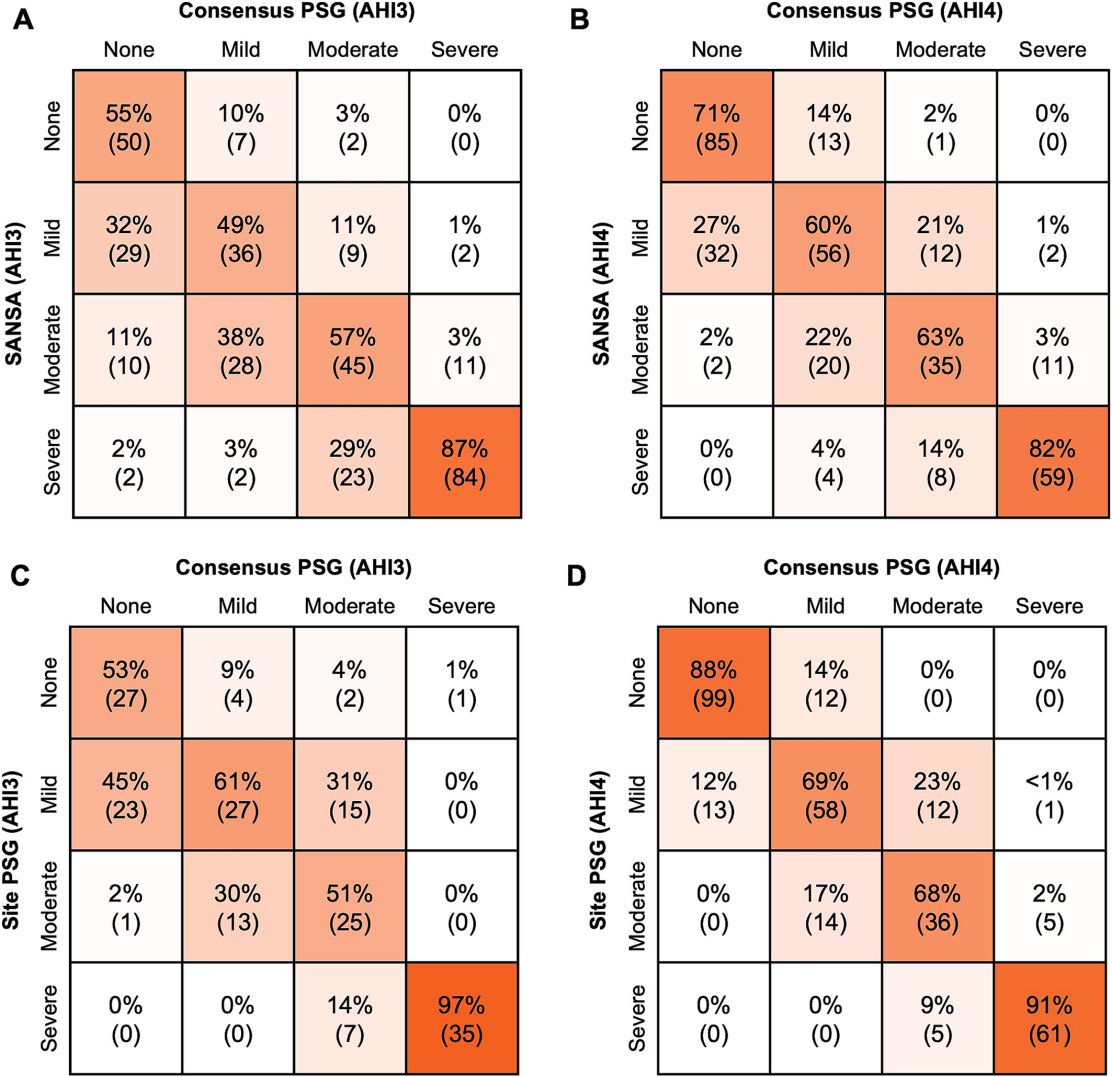
SANSA TST Correlation and Bland–Altman comparison against PSG. TST correlation **(A)** and Bland–Altman **(B)** plots of the SANSA multi-diagnostic device compared to the PSG consensus score. Corresponding plots are also shown for PSG site scores compared to the PSG consensus score **(C,D)**.

Results for TST agreement are detailed in Table 3 and Figure 5. TST correlation with consensus PSG was moderately strong for SANSA (*R* = 0.82) but lower than that of site PSG (*R* = 0.92, *p* < 0.001). Compared to consensus PSG, TST bias was greater and limits of agreement were wider for SANSA versus site PSG. Inter-scorer variability of TST between the three individual core consensus scorers represented by the intraclass correlation coefficient was 0.88. Re-inclusion of available pre-specified exclusions resulted in comparable TST correlation and wider limits of agreement (Supplementary Table S2).

**Table 3:** TST endpoints.

	SANSA	Site PSG
Correlation, R	0.82 (0.78, 0.85)	0.92 (0.90, 0.93)^*^
Bias	20.4 (14.9, 26.0)	5.4 (1.6, 9.3)
Lower LoA	−81.3 (−90.9, −71.7)	−66.4 (−73.2, −59.7)
Upper LoA	122.2 (112.6, 131.7)	77.4 (70.6, 84.2)

This table displays TST-related endpoints and their confidence intervals for SANSA and site PSG. An asterisk indicates significantly greater performance (z-test on Fisher z-transformed correlation coefficients). LoA, limit of agreement; PSG, polysomnography; TST, total sleep time.

### 3.4 Sleep/wake classification

The accuracy of epoch-level sleep/wake classification measured by SANSA against consensus PSG was 87.2% (95% CI: 87.0, 87.5%). The sensitivity and specificity to detect sleep epochs was 95.0% (95% CI: 94.8, 95.1%) and 62.7% (95% CI: 61.9, 63.5%).

## 4 Discussion

These results build on previous work by validating the diagnostic performance of SANSA in a large, diverse, and prospective population. SANSA’s close agreement with PSG combined with its simple, single-point-of-contact design and capability to detect comorbid cardiovascular disorders positions it as an efficient and versatile solution to diagnose and monitor OSA.

SANSA provided acceptable diagnostic performance across all common OSA severity criteria, which is essential to maintain flexibility in current clinical practice given differences between payor criteria and treatment protocols at varying severities. In this study, SANSA classified moderate-to-severe OSA with high sensitivity and specificity using Rule 1B, which was comparable to participating site PSG scores and maintained from SANSA’s previous development results. Moderate-to-severe classification performance decreased slightly with Rule 1A for both SANSA and participating site PSG scores, but remained clinically acceptable and consistent with the performance of other HSATs (Sensitivity: 78–91%, Specificity: 62–76%) (25, 31–33). For mild, moderate, or severe OSA, SANSA and participating site PSG scores maintained high sensitivity but lost specificity for both scoring criteria, though performance remained consistent with or greater than other HSATs for both Rule 1A (Sensitivity: 91–97%, Specificity: 35–75%) (31, 32) and Rule 1B (Sensitivity: 92–95%, Specificity: 64–80%) (25, 34). Consistent with previous reports, this reduced performance for SANSA and participating site PSG using Rule 1A was largely attributed to greater interscorer variability in PSG scoring compared to Rule 1B due to the former including arousals for hypopnea scoring (25, 35, 36).

SANSA’s TST and sleep / wake classification performance were also consistent with reports for other HSAT and actigraphy devices (25, 37–39). As with all sleep monitoring technologies, higher sensitivity to sleep than specificity naturally contributes to better TST agreement as sleep efficiency increases. This trend was observed in the current study for both SANSA and site PSG compared to consensus PSG (data not shown) and aligns with AASM clinical practice guidelines to use PSG in patients with severe insomnia and other conditions associated with poor sleep efficiency (19). Generalizability and transportability of these results across sleep medicine practices are supported by the large and diverse enrollment across seven geographically distributed sites in both academic and community settings. PSG records were collected using site-specific PSG protocols and were scored by three independent scorers for representative consensus scoring. Participants were well represented across age, sex, race, BMI, and skin tone, and included common OSA comorbidities of cardiovascular, respiratory, endocrine and other sleep disorders. Such high degree of study design diversity exceeds that reported by other unattended diagnostic technologies (25, 31, 33, 34, 40). Comparable operational characteristics after reincluding pre-specified exclusions further support stable performance across diverse conditions, remaining consistent with or exceeding other HSATs.

In addition to clinically acceptable diagnostic performance, SANSA’s patch-based form could streamline diagnostic workflows to better manage increasing patient volumes. Its single point of contact on the chest could simplify application and patient training—removing the need for multiple belts, wires, tubing, fingernail clipping, and nail polish removal. Providing a non-digit site for oximetry could also represent a useful alternative for patients with vascular insufficiency (e.g., Raynaud’s). Its Holter-like design is also familiar to referring cardiologists and primary care physicians, which could encourage interdisciplinary collaborations to identify undiagnosed OSA. Given the strong association between OSA, atrial fibrillation, and heart failure (41, 42)—with studies indicating that OSA can increase the risk of developing atrial fibrillation by up to four-fold and contribute to the progression of HF (43, 44)**—**SANSA’s multi-diagnostic capabilities align with the growing need to identify and manage OSA alongside its common comorbidities. In particular, the ability of SANSA to record ECG could support simultaneous detection and assessment of atrial fibrillation and other arrhythmias, similar to PSG and Holter monitors. The potential to measure hemodynamic parameters associated with systolic and diastolic dysfunction further support its potential use in assessing heart failure progression. For example, both pre-ejection period (the time between electrical activation of the heart marked by the R-wave and aortic valve opening) and diastolic filling time (the time between mitral valve opening and closure) may predict heart failure progression in certain HF populations (18, 45). The ability to simultaneously monitor and diagnose these multiple comorbid conditions from a single point of contact device worn during sleep could significantly enhance patient care, allowing for earlier detection and more comprehensive management of at-risk populations.

This validation study was carefully designed to avoid common pitfalls in sleep technology performance evaluations, including challenges with AHI rules, selection of optimal AHI cutoffs, and selective statistical reporting (48). By clearly defining and using both Rule 1A and 1B criteria and reporting results across standard AHI thresholds (≥5, ≥15, and ≥30 events/h), this study provides a transparent and comprehensive evaluation of SANSA’s performance. Furthermore, the inclusion of a large, diverse patient cohort—spanning across age, sex, race, skin tone, and BMI—ensured minority representation in device validation. However, this study does have important limitations that should be considered when interpreting this work. This study was conducted entirely in an in-lab PSG setting during a single night, which has been associated with lower sleep efficiency and the first night effect (46, 47). Enrollment in this setting was also logistically constrained by coordinator and device availability, a common operational challenge in conducting clinical studies. Further investigation is warranted in the home setting and across multiple nights. Comparison between participating site and consensus PSG scores was also limited by the availability of Rule 1A and Rule 1B scores due to payer-motivated local scoring policies at each clinical site.

In conclusion, SANSA demonstrated reliable diagnostic accuracy for OSA, closely aligning with PSG results while offering a less obtrusive, single-point-of-contact design. Its capability to capture multiple physiological signals and provide total sleep time to calculate the AHI supports its use as a comprehensive tool for diagnosing OSA and detecting cardiovascular comorbidities, enhancing clinical and home-based diagnostics. These findings support SANSA’s role as an efficient addition to routine clinical practice, improving diagnostic workflows and patient outcomes in OSA management.

## Data Availability

The raw data supporting the conclusions of this article will be made available by the authors, without undue reservation.
