# Supplementary material for: Polysomnography validation of SANSA to detect obstructive sleep apnea
Source: Front Neurol. 2025 Jun 16;16:1592690. doi: 10.3389/fneur.2025.1592690 (PMC12206633; doi:10.3389/fneur.2025.1592690)
Supplement: Supplementary file 1 [file Table_1.docx]

**Table S1. AHI endpoints with and without available pre-specified exclusions**

|  |  | 3% Rule |  | 4% Rule |  |
| --- | --- | --- | --- | --- | --- |
|  |  | As reported | All available data | As reported | All available data |
| Correlation, R |  | 0.90 (0.87, 0.92) | 0.87 (0.85, 0.90) | 0.91 (0.89, 0.93) | 0.90 (0.88, 0.92) |
| Bias |  | 1.8 (0.6, 3.0) | 2.1 (0.8, 3.3) | 0.3 (-0.8, 1.3) | 0.5 (-0.6, 1.5) |
| Lower LoA |  | -20.4 (-22.5, -18.1) | -22.1 (-24.3, -19.9) | -18.9 (-20.7, -17.1) | -19.8 (-21.7, -18.0) |
| Upper LoA |  | 23.9 (21.8, 26.0) | 26.2 (24.0, 28.4) | 19.4 (17.6, 21.2) | 20.7 (18.9, 22.5) |
| Cutoff AHI 5 | Se | 0.96 (0.93, 0.98) | 0.96 (0.93, 0.98) | 0.94 (0.90, 0.96) | 0.93 (0.89, 0.96) |
|  | Sp | 0.55 (0.44, 0.65) | 0.50 (0.40, 0.61) | 0.71 (0.62, 0.79) | 0.68 (0.60, 0.76) |
|  | PPV | 0.85 (0.81, 0.89) | 0.84 (0.79, 0.88) | 0.86 (0.81, 0.90) | 0.84 (0.79, 0.88) |
|  | NPV | 0.85 (0.73, 0.93) | 0.82 (0.70, 0.91) | 0.86 (0.77, 0.92) | 0.83 (0.75, 0.90) |
|  | Acc | 0.85 (0.81, 0.89) | 0.84 (0.80, 0.87) | 0.86 (0.82, 0.89) | 0.84 (0.80, 0.88) |
| Cutoff AHI 15 | Se | 0.93 (0.88, 0.96) | 0.92 (0.87, 0.96) | 0.88 (0.81, 0.93) | 0.85 (0.78, 0.90) |
|  | Sp | 0.74 (0.67, 0.81) | 0.70 (0.63, 0.77) | 0.87 (0.82, 0.91) | 0.85 (0.80, 0.89) |
|  | PPV | 0.80 (0.73, 0.85) | 0.77 (0.71, 0.82) | 0.81 (0.73, 0.87) | 0.77 (0.69, 0.83) |
|  | NPV | 0.90 (0.84, 0.95) | 0.90 (0.83, 0.94) | 0.93 (0.88, 0.96) | 0.90 (0.86, 0.94) |
|  | Acc | 0.84 (0.79, 0.88) | 0.82 (0.77, 0.85) | 0.88 (0.84, 0.91) | 0.85 (0.81, 0.88) |
| Cutoff AHI 30 | Se | 0.87 (0.78, 0.93) | 0.83 (0.74, 0.90) | 0.82 (0.71, 0.90) | 0.79 (0.68, 0.88) |
|  | Sp | 0.89 (0.84, 0.93) | 0.87 (0.83, 0.91) | 0.95 (0.92, 0.97) | 0.95 (0.92, 0.97) |
|  | PPV | 0.76 (0.67, 0.83) | 0.72 (0.63, 0.80) | 0.82 (0.71, 0.90) | 0.80 (0.70, 0.89) |
|  | NPV | 0.94 (0.90, 0.97) | 0.93 (0.89, 0.96) | 0.95 (0.92, 0.97) | 0.95 (0.91, 0.97) |
|  | Acc | 0.88 (0.84, 0.91) | 0.86 (0.82, 0.89) | 0.92 (0.89, 0.95) | 0.92 (0.88, 0.94) |

This table displays AHI-related endpoints and their 95% confidence intervals (Clopper-Pearson) for SANSA using the 3% rule and the 4% rule in the as-reported population (N=340) and the population containing all available data (N=375). No significant differences were observed between populations for the same scoring rule (z-test on Fisher z-transformed correlation coefficients and Fisher’s exact test for proportions). Acc = accuracy; AHI = apnea–hypopnea index; NPV = negative predictive value; PPV = positive predictive value; PSG = polysomnography; Se = sensitivity; Sp = specificity.

**Table S2. TST endpoints with and without available pre-specified exclusions**

|  | As reported | All available data |
| --- | --- | --- |
| Correlation, R | 0.82 (0.78, 0.85) | 0.79 (0.75, 0.83) |
| Bias | 20.4 (14.9, 26.0) | 17.5 (11.7, 23.3) |
| Lower LoA | -81.3 (-90.9, -71.7) | -94.5 (-104.6, -84.4) |
| Upper LoA | 122.2 (112.6, 131.7) | 129.6 (119.5, 139.6) |

This table displays TST-related endpoints and their confidence intervals for SANSA in the as-reported population (N=340) and the population containing all available data (N=375). No significant differences were observed between populations (z-test on Fisher z-transformed correlation coefficients). LoA = limit of agreement; PSG = polysomnography; TST = total sleep time.
